# ADA-GEL composite hydrogel films incorporating mesoporous bioactive glass nanoparticles and silver-doped bioactive glass nanoparticles for biomedical applications

**DOI:** 10.1007/s10856-026-07057-8

**Published:** 2026-05-05

**Authors:** Asimenia Lekidou, Thomas Papatasos, Qaisar Nawaz, Xanthippi Chatzistavrou, Aldo R. Boccaccini

**Affiliations:** 1 Department of Chemical Engineering, Laboratory of Process Systems Engineering, Aristotle University of Thessaloniki, Thessaloniki, Greece; 2 Department of Physics, Aristotle University of Thessaloniki, Thessaloniki, Greece; 3 Department of Materials Science and Engineering, Institute of Biomaterials, University of Erlangen-Nuremberg, Erlangen, Germany

## Abstract

Hydrogels made from natural polymers are widely used in biomedical engineering as they resemble the extracellular matrix. Alginate dialdehyde-gelatin (ADA-GEL) hydrogels are notable for their biocompatibility, biodegradability, and adjustable crosslinking. However, their limited mechanical strength and bioactivity present significant challenges for use in tissue regeneration and wound healing. To overcome these issues, this study explores the addition of 0.1 and 1 wt.% mesoporous bioactive glass nanoparticles (MBGNs) or silver-doped bioactive glass nanoparticles (Ag-BGNs) into ADA-GEL hydrogels to create composite hydrogel films. These nanocomposites were tested in terms of printability, swelling, degradation, antibacterial effects, and in vitro compatibility using MG-63 cells (osteosarcoma cells). MBGNs improved the hydrogels’ osteogenic ability and structural stability. At the same time, Ag-BGNs provided vigorous antibacterial activity, especially against *Staphylococcus aureus* and *Escherichia coli*, without significantly affecting cell viability and morphology. The use of bioactive and antimicrobial nanoparticles in the ADA-GEL matrix offers a promising approach for developing new soft biomaterials for applications such as bone regeneration, wound healing, and implant coatings.

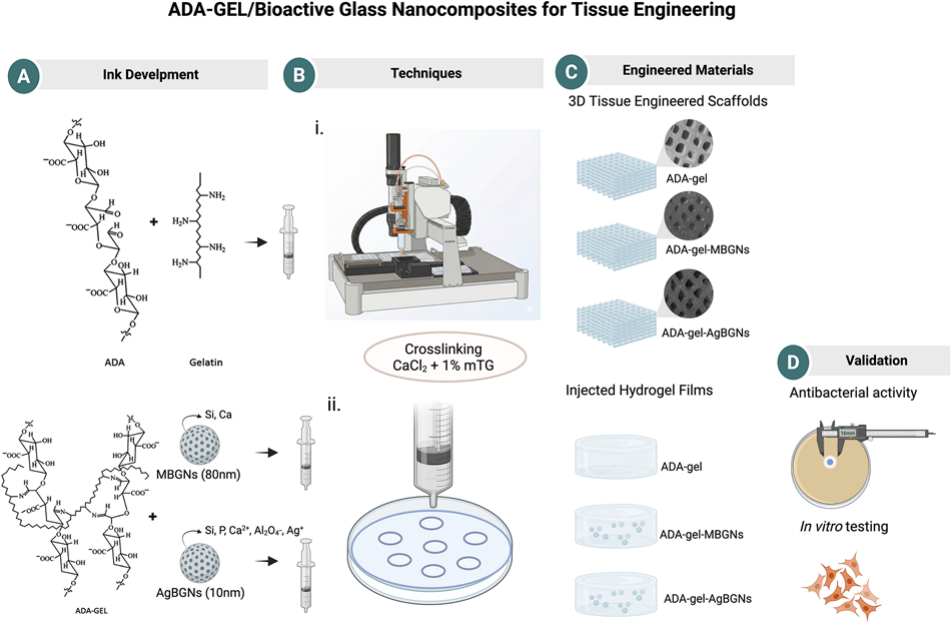

## Introduction

Hydrogels, which are three-dimensional polymeric networks that swell with water, are widely recognized for their ability to mimic the highly hydrated extracellular matrix (ECM) [1]. These characteristics make them excellent candidates for biomedical applications such as tissue engineering, drug delivery, and wound healing. Among various hydrogels, those made from natural polymers like alginate and gelatin have attracted significant attention [2, 3].

Alginate, a polysaccharide derived from brown algae, is known for its shear-thinning behavior, which is a crucial property for extrusion-based bioprinting [2]. It also boasts biocompatibility and non-toxic degradation products. However, pure alginate lacks cell adhesion motifs, limiting its ability to support cellular functions [4].

To overcome this limitation, alginate has been chemically modified to create alginate dialdehyde (ADA), which introduces reactive aldehyde groups [5]. When combined with gelatin (GEL), a denatured collagen derivative that contains RGD (arginine-glycine-aspartic acid) sequences to promote cell adhesion, the resulting ADA-GEL hydrogels exhibit improved biofunctionality [6, 7]. This is due to Schiff base reactions between the aldehyde groups of ADA and the amine groups of gelatin, which form covalent crosslinks and establish a stable, cell-interactive network. Additionally, the mechanical and degradation properties of ADA-GEL hydrogels can be fine-tuned [8]. They can undergo further crosslinking through enzymatic (e.g., microbial transglutaminase) or ionic (e.g., calcium ions) methods, providing precise control over their properties [2].

Despite these advancements, natural hydrogels like ADA-GEL still face challenges, including inherent mechanical weakness and limited bioactivity, especially in load-bearing or regenerative applications [8]. To address these issues, the incorporation of inorganic nanoparticles has emerged as a promising strategy. These nanoparticles can serve as rheological modifiers to enhance printability and structural integrity, as well as functional agents that introduce bioactive and/or antimicrobial properties.

In this study, the integration of two types of inorganic bioactive nanoparticles into ADA-GEL hydrogels was investigated. Mesoporous Bioactive Glass Nanoparticles (MBGNs) and Silver-doped Bioactive Glass Nanoparticles (Ag-BGNs) (0.1 wt.%) were studied. MBGNs, in the 70 SiO₂ - 30 CaO mole % system, were synthesized via the solution-gelation (sol-gel) method, have a high surface area and pore volume, enabling ion exchange and apatite formation. These nanoparticles have been shown to enhance osteogenic differentiation and promote hydroxyapatite nucleation, which are crucial for bone tissue engineering [9].

On the other hand, Ag-BGNs incorporate silver ions into the glass matrix, providing the hydrogel with strong antibacterial properties without compromising biocompatibility [10]. The controlled release of silver ions during glass degradation results in a sustained antimicrobial effect that is particularly effective against pathogens like *Staphylococcus aureus*. Furthermore, Ag-BGNs have been shown to stimulate the proliferation and differentiation of osteoprogenitor cells, thereby extending their potential in tissue regeneration [10].

The incorporation of MBGNs and Ag-BGNs into ADA-GEL enhances not only the hydrogel’s bioactivity but also imparts antimicrobial properties, addressing key challenges in tissue engineering and wound healing [11]. The resulting ADA-GEL nanocomposite hydrogel films combine the ECM-mimicking and tunable properties of natural hydrogels with the regenerative and protective functionalities of advanced bioactive nanoparticles [12]. While previous studies have focused on ADA-GEL hydrogels incorporating MBGNs, this study is the first systematic effort to incorporate silver-doped bioactive glass nanoparticles (Ag-BGNs) into the ADA-GEL matrix to specifically leverage their potent antibacterial properties. Using the well-established ADA-GEL/MBGN system as a performance benchmark, we investigated how adding Ag-BGNs at a refined concentration of 0.1 wt.% provides essential protection against pathogens without compromising the scaffold’s 3D printability or cytocompatibility. This work offers a contribution to the field of multifunctional composite hydrogels for 3D printing; additionally, it paves the way for future research to explore the simultaneous incorporation of MBGNs and Ag-BGNs to achieve a synergistic balance between enhanced osteogenesis and improved infection control within a single platform. Such hydrogel films are especially suitable for bone regeneration, infected wound treatment, and implant coatings, applications where tissue integration and infection prevention are vital.

## Materials and methods

### Synthesis of mesoporous bioactive glass nanoparticles (MBGNs)

The materials included Tetraethyl orthosilicate (TEOS), triethyl phosphate (TEP), and calcium nitrate (Ca(NO_3_)_2_ · 4H_2_O), which served as sources of silicon, phosphorus, and calcium. Additionally, ethyl acetate, cetyltrimethylammonium bromide (CTAB), ammonium hydroxide (28%), distilled water (MilliQ), and absolute ethanol were used. All chemicals were of analytical grade and were purchased from Sigma-Aldrich, Germany. The fabrication of MBGNs via micro-emulsion-assisted sol–gel method was previously reported in the literature [13]. Briefly, 1.12 g of CTAB was dissolved in 52 mL of Milli-Q water (ELGA DV 25, PURELAB Option R7BP, UK) under continuous stirring at 37 °C. Once fully dissolved, 16 mL of ethyl acetate was added, and the mixture was stirred for an additional 30 min to form a stable microemulsion. Subsequently, ammonium hydroxide solution was introduced to the system to establish a basic environment (pH ~10), and stirring was continued for 15 min to facilitate TEOS hydrolysis. Following this, 10 mL of TEOS was added to the reaction mixture. 4.6 g of Calcium nitrate tetrahydrate was introduced in varying amounts at 30-min intervals to allow for gradual incorporation into the silica network. The reaction proceeded under stirring for an additional 4 h to ensure complete condensation and particle formation. The resulting colloidal suspension was collected via centrifugation at 4200 rpm for 10 min. The precipitate was washed multiple times with ethanol and deionized water to remove residual surfactant and unreacted precursors. After drying overnight at room temperature, the dried particles were calcined in air at 700 °C for 3 h using a controlled heating rate of 2 °C/min to remove the CTAB template and stabilize the mesoporous structure.

### Synthesis of silver-doped bioactive glass nanoparticles (Ag-BGNs)

The synthesis of Ag-BGNs (SiO_2_ 59.6-CaO 25.5-P_2_O_5_ 5.1-Al_2_O_3_ 7.2-Ag_2_O 2.2 wt%) was performed through a modified Stöber-like protocol involving the preparation of two separate precursor solutions, designated as Solution A and Solution B, as reported in the literature [10]. All reagents were obtained from ChemLab and used without further purification.

Solution A consisted of methanol (CH₃OH), tetraethyl orthosilicate (TEOS, Si(OC₂H₅)₄), triethyl phosphate (TEP, C₆H₁₅O₄P), aluminum nitrate nonahydrate (Al(NO₃)₃·9H₂O, AlNT), silver nitrate (AgNO₃, AgNT), and calcium nitrate tetrahydrate (Ca(NO₃)₂·4H₂O, CaNT). A volume of 41.2 mL of methanol was transferred into a Teflon beaker, and mechanical stirring was initiated at 500 rpm while maintaining the temperature at 25 °C. Subsequently, 5.6 mL of TEOS and 0.46 mL of TEP were added sequentially. Methanol was selected as the solvent to promote the hydrolysis of TEP, enabling both TEOS and TEP to hydrolyze at comparable rates. This synchronization facilitates the homogeneous incorporation of phosphorus (P) into the SiO₂ network before particle nucleation. After 24 h of continuous stirring, 1.14 g of AlNT was added to the mixture. Aluminum nitrate aids in stabilizing silver ions, promoting their incorporation in ionic form. Next, 0.11 g of AgNO₃, pre-ground in a porcelain mortar to enhance solubility, was introduced as the silver source to impart antibacterial properties to the material. Subsequently, 2.78 g of CaNT was added and stirred for 24 h to enable the calcium ions (Ca²⁺) to interact with and become embedded within the forming silica network.

In parallel, Solution B was prepared by mixing 21 mL of distilled water, 31.5 mL of ethanol (EtOH), and 10 mL of ammonium hydroxide (25 wt%) under magnetic stirring for 30 min to ensure homogeneity. This catalytic solution was then added to Solution A to initiate condensation and gelation.

After 24 h, the sol turned white and acquired a viscous consistency, indicating the formation of nanoparticles. The resulting dispersion was centrifuged at 2000 rpm for 3 min using polypropylene tubes to isolate the solid phase. The precipitate was washed with 25 mL of ethanol to remove residual calcium and any surface impurities. The recovered material was transferred to a crucible suitable for thermal treatment. The thermal processing was carried out using the following profile:

- Heating from 25 °C to 60 °C at 5 °C/min and holding for 6 h
- Heating from 60 °C to 700 °C at 2 °C/min and holding for 2 h
- Cooling from 700 °C to 25 °C at 5 °C/min

After calcination, the material was ground into a fine powder. To remove any unincorporated Ca²⁺ ions loosely bound to the particle surface, the powder was washed twice with ethanol. Finally, the material was dried at 60 °C for 24 h and ground again to yield the final Ag-BGN powder.

### Synthesis of alginate dialdehyde (ADA)

The materials used include: Sodium alginate (MW 100,000–200,000 g/mol, guluronic acid content 65–70%, approved as a pharmaceutical excipient) and gelatin (Bloom 300, Type A, porcine skin, suitable for cell culture), both purchased from Sigma-Aldrich, Germany. Sodium metaperiodate and calcium chloride dihydrate (CaCl₂·2H₂O) were purchased from VWR International, Belgium. Microbial transglutaminase (mTG) was acquired from Ajinomoto Foods, Europe. For the synthesis, ADA was produced through the oxidation of sodium alginate, following a protocol previously described in the literature [14]. Briefly, 10 g of alginate was dispersed in 50 mL of ethanol (EtOH 99%) and mixed dropwise with an aqueous solution of 9.375 mmol or 12.5 mmol sodium (meta)periodate (NaIO₄), respectively. The entire reaction was carried out in the dark at room temperature. Vigorous stirring for 6 h was followed by adding 10 mL of ethylene glycol to quench any excess periodate (IO₄⁻) ions in the reaction mixture, under stirring for an additional 30 min. Subsequently, each reaction mixture was divided into 50 mL Falcon tubes, centrifuged at 2500 rpm for 5 min in the dark, and the ethanol phase was decanted. The remaining ADA solution was dialyzed (using Spectrum Labs™ Dialysis Membranes, MWCO: 6–8 kDa) against 15 L of MilliQ water for 4 days, with daily water changes. The product was then frozen for at least 24 h, followed by lyophilization using a freeze dryer (Christ Alpha 1-4LD, Christ Gefriertrocknungsanlagen, Osterode am Harz, Germany), resulting in dry, oxidized ADA.

### Nanocomposite hydrogel films made of ADA-Gel and NPs

ADA (alginate dialdehyde) and gelatin (GEL) (from porcine skin, Type A) were prepared in equal volumes in Dulbecco’s Phosphate Buffered Saline (DPBS) (−Ca²⁺, −Mg²⁺) following the published protocol [15]. Briefly, 5% (w/v) solutions of lyophilized ADA and 7.5% (w/v) of GEL were separately dissolved in DPBS and continuously stirred at 37 °C until homogeneous solutions formed. Both solutions were passed through 0.45 µm syringe filters for ADA and 0.22 µm syringe filters for GEL, respectively. The filtered solutions were mixed in a 1:1 ratio under continuous stirring to facilitate crosslinking between ADA and GEL, ultimately forming 5% (w/v) ADA-7.5% (w/v) GEL hydrogels. To prepare the solution with MBGNs and AgBGNs, the filtered ADA solution was added to a beaker. Then, 0.1 and 1% (w/v) MBGNs and 0.1 and 1% (w/v) AgBGNs were added to the ADA solution over approximately 15 min of stirring. To ensure uniform dispersion of the particles, the mixture was ultrasonicated for 15 min. Afterward, the filtered gelatin solution was added to the ADA-MBGNs mixture in a 1:1 ratio and stirred until homogenized. The particles were then sterilized in an oven at 120 °C for two h. Subsequently, the ADA solution was pipetted into the particles and stirred in a water bath at 37 °C inside the sterile bench.

**Fig. 1:**
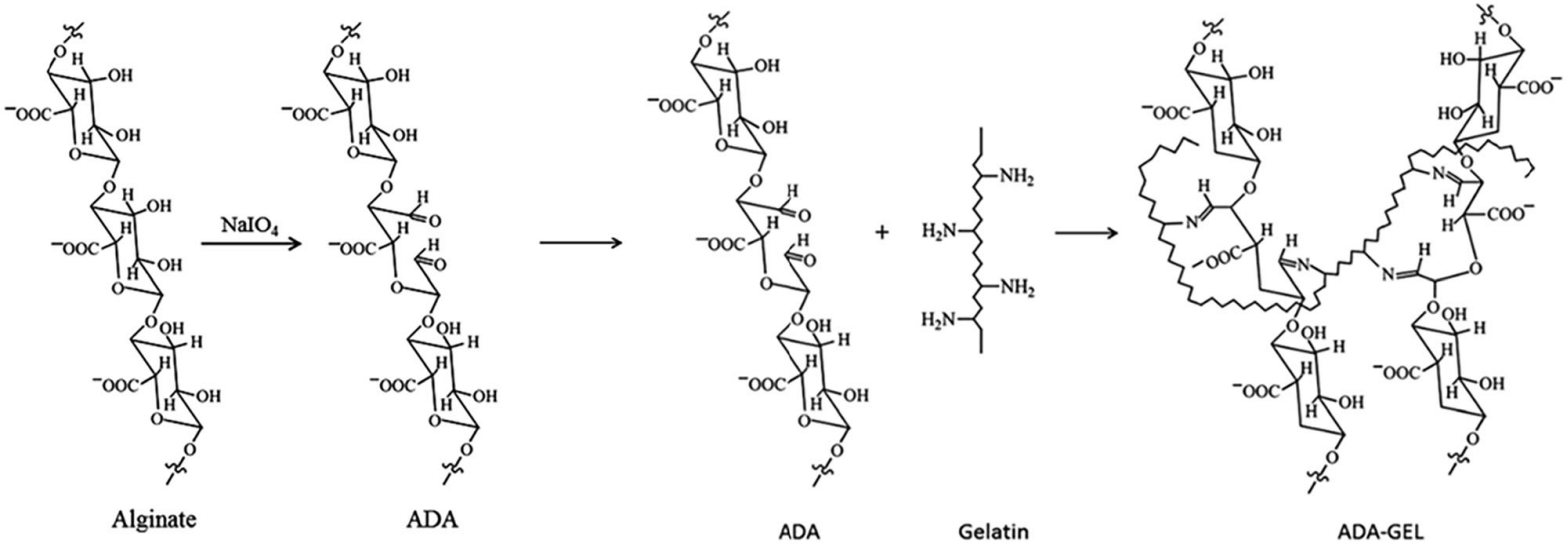
Schematic presentation of ADA synthesis from alginate by periodic oxidation, followed by crosslinking of ADA and gelatin, and possible coordination of ADA molecules with gelatin chain [14, 39]

For preparing the films, 250 μL of sterile filtered hydrogel precursor solution (ADA-GEL/ADA-GEL-MBGNs/ADA-GEL-AgBGNs) was poured into silicone molds (diameter = 12 mm, height = 2 mm), placed in petri dishes, and then cooled for 15 min in the fridge at 4 °C to accelerate the thermal gelation of gelatin. This cooling helps stabilize the surface of the films. Next, a crosslinking solution containing 1% (w/v) mTG in 1 M CaCl_2_ was added to the hydrogel-filled molds for 15 min. After removing the molds from the fridge, a spatula was used to ensure the crosslinker solution penetrated from all sides during this period. The films were then carefully detached from the silicone molds. Additionally, the films were separated from the bottom of the petri dishes, allowing them to float in the crosslinking solution so that the bottom surfaces could be crosslinked. After about 15 min, Hank’s Balanced Salt Solution (HBSS) with (+) Ca^2+^ and (+) Mg^2+^ was used to halt the crosslinking reaction and rinse the films twice. The different groups and their systems are outlined in Table 1. The overall reaction scheme is depicted in Fig. 1.

**Table 1:** Fabricated groups

Names	Systems
ADA-GEL (control)	5% w/v ADA-7.5% w/v GEL
ADA-GEL-0.1%AgBGNs	5% w/v ADA-7.5% w/v GEL /0.1% w/v AgBGNs
ADA-GEL-0.1%MBGNs	5% w/v ADA-7.5% w/v GEL /0.1% w/v MBGNs

### In vitro degradation and swelling studies

The weight changes of ADA-GEL and the composite hydrogels were examined through in vitro incubation of crosslinked hydrogel films under physiological conditions. The films were prepared as previously described (part “Nanocomposite hydrogel films made of ADA-Gel and NPs”). The samples (*n* = 6) were incubated in 6-well plates (Sarstedt, Nümbrecht, Germany) containing 5 mL DMEM at 37 °C with 5% CO₂ and 95% relative humidity. To weigh the samples, the films were placed into previously weighed cell strainers (Cell Strainer, Corning®, NY, US). The medium was changed twice weekly to mimic cell culture conditions and prevent accumulation of GEL and other degradation products. All samples were weighed at times 0, 1, 2, 3, 4, 5, 6 h, and on days 1, 3, 7, 14, and 21. Before weighing, cell strainers with films were dried to remove excess medium. The weight change of the hydrogel films was evaluated using a wet-mass-to-wet-mass comparison to accurately simulate the real-time behavior of the biomaterial in a physiological environment. By calculating weight changes relative to the initial wet mass (mi), the study accounts for the continuous hydrated state that films maintain during clinical application, where the material does not return to a dry state. Swelling and degradation at each time point were calculated in weight percentage using the following equation:

$$Swelling\left[wt \% \right]\,or\,Degradation\left[wt \% \right]=\frac{{m}_{{c}}-{m}_{{i}}}{{m}_{{i}}}$$

Where:

*m*_*c*_ = the current mass at the specific time point.

*m*_*i*_ = the initial wet mass of the film before incubation in DMEM.

### Fabrication of hydrogel scaffolds

ADA-GEL constructs (with or without NPs) were created using a pneumatic extrusion-based 3D printer (BioScaffolder 3.1, GeSiM). The square-shaped constructs, 10 mm long, were printed with four layers along the z-axis. The hydrogel was loaded into an ink cartridge connected to a compressed air source, which drove the material through a 25 G (250 μm) nozzle to print in six-well tissue culture plates (VWR, Germany) layer by layer. The applied air pressure was 120 kPa, and the printing speed was 10 mm/s. The parameters were initially adopted from the established protocol by Monavari et al. for ADA-GEL/MBGN systems [16]. These settings were then confirmed through preliminary “dose-response” trials aimed at achieving continuous filament extrusion at the lowest possible pressure to minimize mechanical shear stress on the macromolecular network. After printing, 0.1 M CaCl_2_ and 1% mTG solution were used to induce ionic gelation (crosslinking) of the hydrogel. After 30 min, the samples were thoroughly washed with Hank’s Balanced Salt Solution (HBSS, Gibco®, Darmstadt, Germany) to remove any remaining CaCl_2_.

### Antibacterial testing

The agar disk diffusion test was performed on the fabricated groups of Table 1. First, 25 mL of nutrient agar was poured into petri dishes, then 25 μL of overnight-cultured bacterial strains, *Escherichia coli* (*E. coli*) and *Staphylococcus aureus* (*S. aureus*), with an optical density of 0.015 (OD_600_), were spread over the plates to form a uniform bacterial lawn. The tested hydrogel films were sterilized under UV light for 45 min on each side. Afterward, hydrogel films were placed on the agar plates and incubated at 37 °C for 24 h. After 24 h, all cultured agar plates were observed under an optical microscope for inhibition zone measurements.

### In vitro cytotoxicity

To assess the cytotoxicity, hydrogel films were fabricated as reported in Section “Nanocomposite hydrogel films made of ADA-Gel and NPs”. Then, the films with Human osteoblastic MG-63 cells were seeded in DMEM (Sigma-Aldrich, St. Louis, MO) containing 10% heat-inactivated fetal bovine serum (FBS) and 1% penicillin-streptomycin antibiotics (Sigma-Aldrich, St. Louis, MO) in a humidified incubator at 37 °C with 5% CO_2_. Before starting the experiment, all films were sterilized under UV light for 1 h. Cells at a density of 5 × 10^4^ cells/mL of medium in each well were seeded on top of the films and in the middle of the well in the control plate (without films). After 24, 72, and 168 h of seeding, 0.4 mL of WST-8 solution was added to the wells containing the samples, and the plates were incubated for 3 h. In this assay, metabolically active cells reduce the tetrazolium salt (WST-8) to formazan, which diffuses into the medium and produces a visible color change. The absorbance maximum of the dye at 450 nm, measured via spectrophotometry, serves as an indirect indicator of mitochondrial activity and, consequently, cell viability [17]. After incubation, 100 μL of the culture sample was taken from each well and transferred to a 96-well plate to evaluate cell viability using absorbance measurements with a microplate reader following the WST-8 protocol. Each experiment was performed in five replicates under sterile conditions, and the mean values and standard deviations were reported.

### Fluorescence assay

For preparing samples for fluorescence microscopy analysis, Calcein solution (4 µL of Calcein per mL of PBS) was added to cover the samples, then the plate was covered with aluminum foil and incubated for 45 min. Calcein-AM, a non-fluorescent, cell-permeant dye, is enzymatically converted inside living cells into a fluorescent compound, providing insights into cell shape and membrane integrity [18, 19]. Inside a hood under non-sterile conditions, the Calcein solution was removed, and the samples were washed with PBS. Fluo-Fix Fixing Solution (37% Formaldehyde/90 mL of PBS) was then added, and the samples were left for 30 min. Before removing the Fixing Solution, 4′, 6-diamidino-2-phenylindole (DAPI) solution (1 µL of DAPI per 1 mL of PBS) was added to cover the samples. DAPI, a blue-fluorescent stain, binds to DNA and specifically marks the nuclei [18, 19]. The samples were covered with aluminum foil and left at room temperature for 40 min. After removing the DAPI solution, the samples were washed with PBS and kept in PBS until analysis under a fluorescence microscope (Axio Scope A.1, Carl Zeiss, Germany).

### Statistical analysis

Data analyses were conducted using one-way and two-way ANOVA followed by Tukey’s multiple comparison test in OriginLab software (OriginLab, 2021, USA). The statistical significance was determined based on the following *p* value thresholds: *p* < 0.05 (*), *p* < 0.01 (**), *p* < 0.001 (***), and *p* < 0.0001 (****). The data are presented as mean ± standard deviation (SD). All experiments were performed with three independent nanoparticle synthesis batches (*n* = 3). For each batch, all analyses were conducted in triplicate to ensure reproducibility and statistical reliability. The degradation/swelling studies were conducted on *n* = 6 samples per group.

## Results

### Morphological and structural characterization

**Fig. 2:**
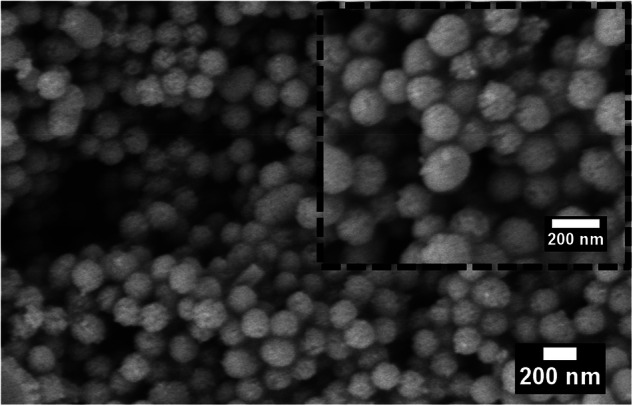
SEM images of MBGNs showing their spherical morphology and agglomerations

Scanning electron microscopy (SEM) was used to assess the size and morphology of the particles. As shown in Fig. 2, MBGNs have spherical, monodisperse particles in the size 80 to 90 nm. Meanwhile, Ag-BGNs produced using a modified Stöber method, as briefly described earlier, are spherical, dense nanoparticles with a size of around 10 nm, as reported by Pajares et al. [10].

**Fig. 3:**
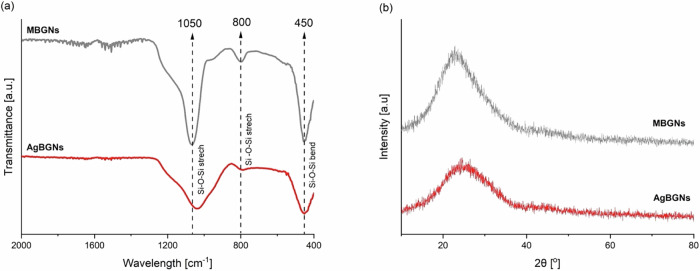
**a** FTIR spectra of MBGNs and AgBGNs confirm the characteristic bioactive glass transmittance peaks, and **b** XRD patterns indicate the amorphous structure of both MBGNs and AgBGNs

Fourier transform infrared (FTIR) spectroscopy was used to study the characteristic bonds of the synthesized MBGNs. The figure shows that the chemical bonding of the silica networks in the MBGNs remained consistent. The characteristic transmittance peaks of the bioactive glass were observed, as shown in Fig. 3a. A peak around 460 cm⁻¹ corresponds to the Si-O-Si bending vibration [20]. Peaks at 800 and 1000 cm⁻¹ are attributed to symmetric and asymmetric Si-O-Si stretching modes, respectively, indicating the presence of SiO₄ tetrahedra, the fundamental units of the glass network [13]. The FTIR spectra of Ag-BGNs revealed that peaks at 470 cm⁻¹ originate from the asymmetric bending vibration of the Si-O-Si bond [11]. Additionally, in the 580–625 cm⁻¹ region, an asymmetric bending vibration is observed, attributed to the P-O bond [16]. The peak at 790 cm⁻¹ corresponds to the stretching vibration of the Si-O-Si bond, while the spectrum shows a broadening of the peak between 900 and 1050 cm⁻¹ [21]. The shoulder at 900 cm⁻¹ is attributed to Si-O-NBO bonds, where NBO refers to non-bridging oxygen groups [21]. This shoulder indicates the successful incorporation of Ca²⁺, Ag⁺, and Al₂O₄⁻ ions, which act as network modifiers of the silicate structure, causing the breaking of Si-O-Si bonds [21].

The XRD patterns (Fig. 3b) of both MBGNs and Ag-BGNs confirm that the synthesized nanoparticles have an amorphous structure, due to the lack of any characteristic peak and the presence of a broad halo instead.

The oxidation of alginate and the incorporation of gelatin were previously studied in detail by Sarker et al. and Karakaya et al. [14, 22]. In this foundational work, the chemical transformation was rigorously confirmed by FTIR and NMR spectroscopy, specifically identifying the formation of hemiacetal carbons corresponding to the generated aldehyde groups at 92.2 ppm [14]. By adopting this validated approach, the degree of oxidation and subsequent covalent crosslinking kinetics with gelatin (GEL) via Schiff base formation were maintained consistent with established physicochemical and microstructural standards. Additionally, the loading of MBGNs within ADA-GEL hydrogels was previously confirmed by Zhao et al. through FTIR spectra, specifically by the bending and stretching vibrations associated with the silica network in the MBGNs (450, 800, and 1050 cm^−1^), along with the characteristic absorption bands of ADA-GEL (1621, 1557, and 1404 cm^−1^) [18]. Likewise, the composite hydrogels of Ag-BGNs within ADA-GEL are also anticipated to present the vibration of the characteristic bonds of both systems.

### Swelling and degradation studies

**Fig. 4:**
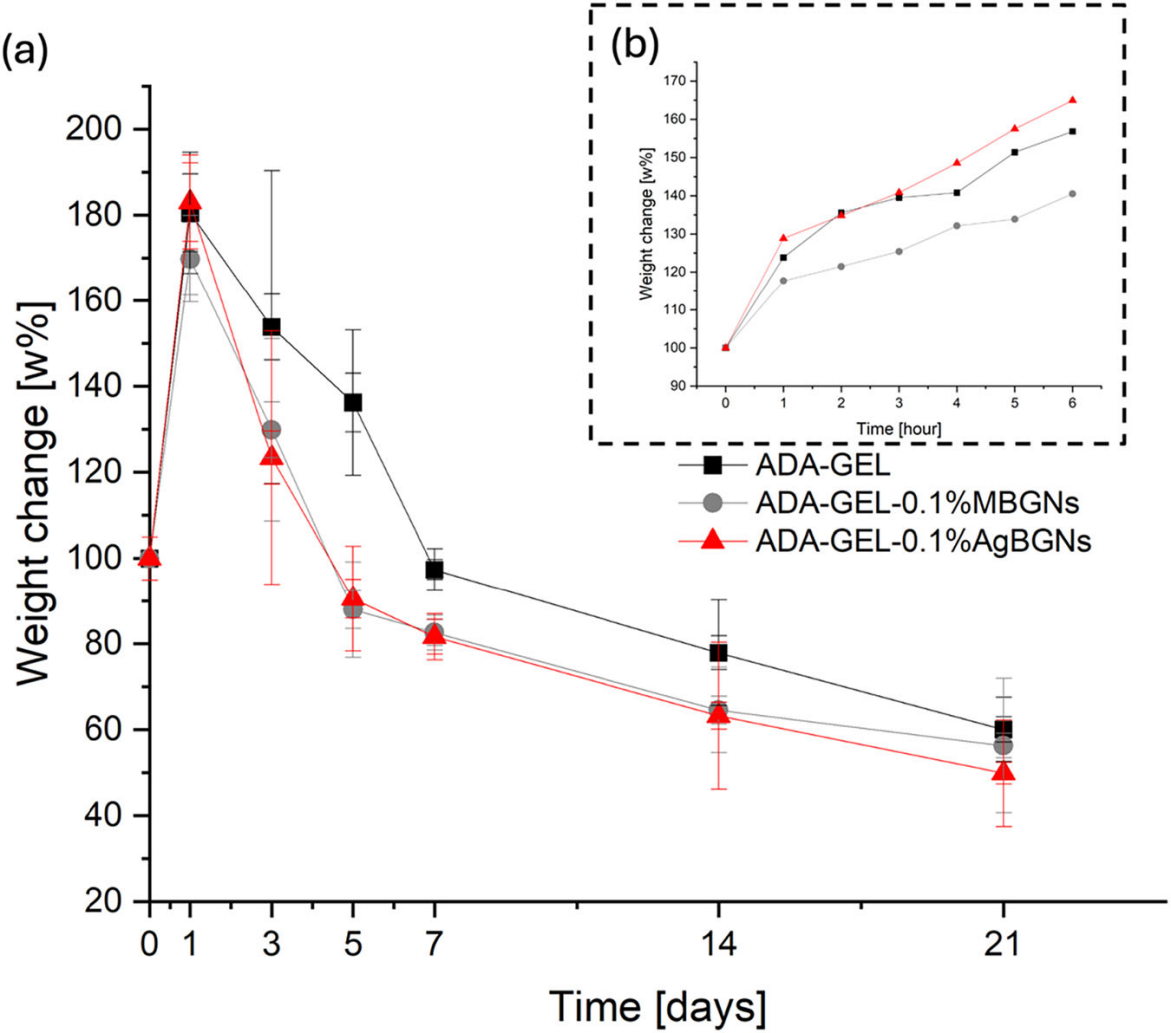
**a** Degradation/swelling study of pure ADA-GEL films (control) and ADA-GEL-MBGNs/AgBGNs films with 0.1% (w/v) MBGNs/AgBGNs concentration after 21 d of incubation, and **b** 6 h of incubation

The weight loss of pure ADA-GEL hydrogel and composite ADA-GEL-0.1%w/v MBGNs, ADA-GEL-0.1%w/v Ag-BGNs hydrogels was measured over 21 days of incubation in DMEM medium (Fig. 4a). The time-dependent weight changes can be divided into two stages. In the first stage, Fig. 4b, the hydrogel samples swelled and gained weight due to absorbing the medium. Regardless of the composition, the weight of all samples increased significantly after 1 day of incubation and reached a peak on day 3. The second stage, which lasted until day 21, involved weight reduction caused by the release of uncross-linked polymer segments and particles, as well as the dissociation of the entire structure.

### 3D printing

**Fig. 5:**
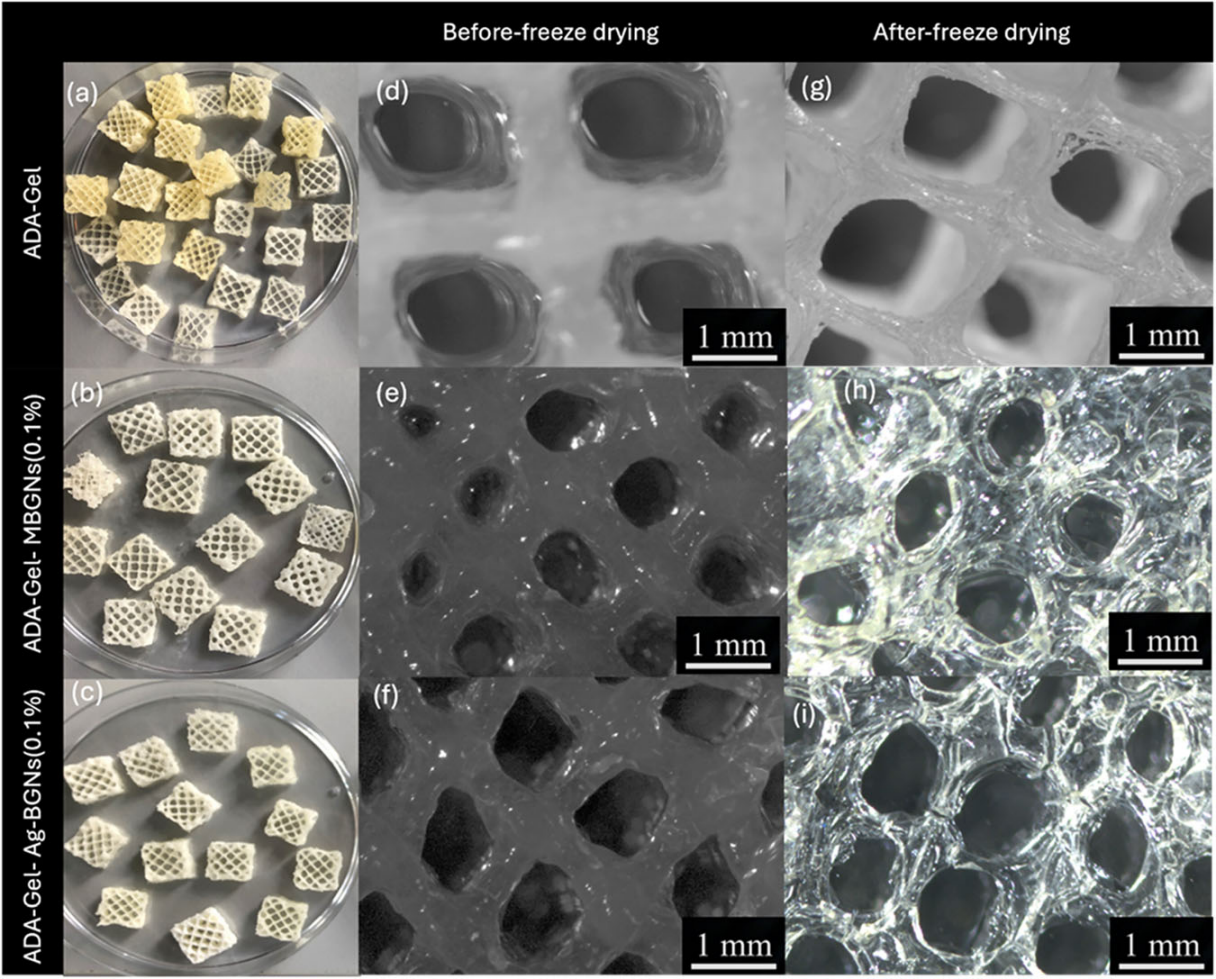
Light microscopy images of 3D extrusion printed 4-layered scaffolds of all investigated ink compositions. Scale bar: 1 mm ADA-GEL (control), ADA-GEL-0.1%w/v MBGNs, and ADA-GEL-0.1%w/v Ag-BGNs. **a**–**c** Overall image of the samples. **d**–**f** Microscopy images of the scaffold after crosslinking. **g**–**i** Microscopy images of the scaffolds after freeze-drying

The 3D printability of the synthesized groups was tested. The scaffolds obtained are shown in Fig. 5 and represent a proof-of-concept for the structural feasibility of printing these specific nanocomposites. In the absence of direct rheological measurements, the assessment of viscosity and printability relied on qualitative morphological criteria, including filament stability during extrusion and preservation of the grid-like architecture without layer fusion or structural drooping. Incorporating 0.1% (w/v) NPs improved the stiffness and grid shape accuracy of scaffolds. The presence of silicate bioactive glass (BG) particles enhanced the viscosity and mass flow of ADA-GEL hydrogels over time through ionic crosslinking mechanisms driven by calcium ions released from the BG nanoparticles [16, 23]. The absence of structural distortion indicates better shape fidelity with NPs concentrations, suggesting that the nanoparticles were homogeneously dispersed without compromising the hydrogel matrix. MBGNs can release calcium ions for additional crosslinking, which helps stabilize the hydrogel network at an optimal concentration of 0.1% (w/v). Moreover, incorporating 0.1% (w/v) MBGNs or AgBGNs did not significantly alter the viscosity of the ADA-GEL hydrogel, suggesting homogeneous nanoparticle dispersion and no compromise to the ADA-GEL matrix. This printing attempt demonstrates that ADA-GEL bioinks containing MBGNs or AgBGNs are printable and retain their shape after fabrication.

### Antibacterial testing

**Fig. 6:**
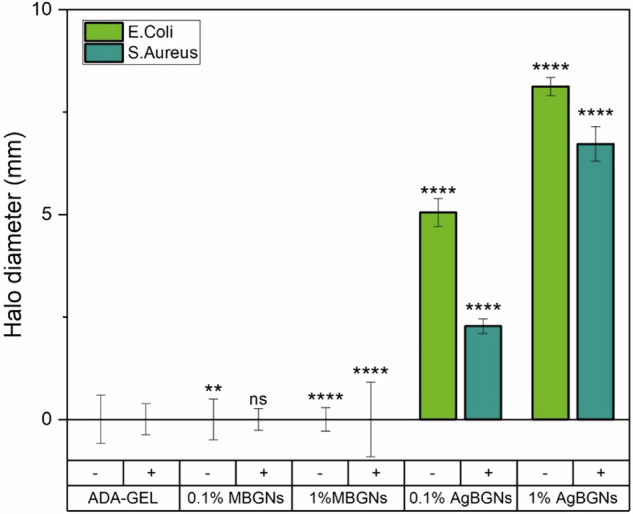
Zone of inhibition for ADA-GEL and the composite hydrogels with MBGNs and AgBGNs in 0.1 and 1% w/w, Values shown are mean and standard deviation (*n* = 3). **p < 0.01; ****p < 0.0001

**Fig. 7:**
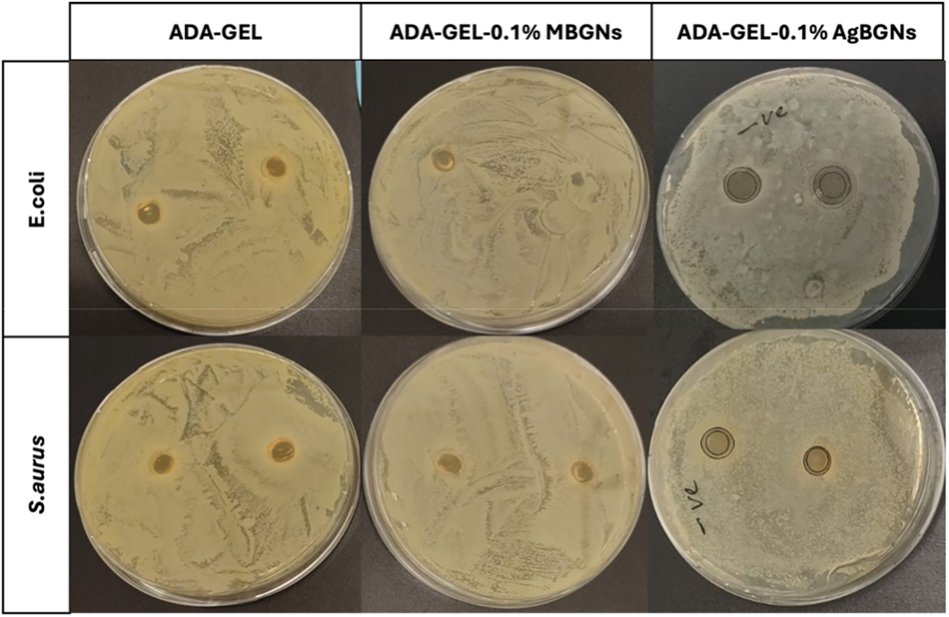
Antimicrobial activity of ADA-GEL and composite films with low concentration of nanoparticles against *E. coli* and *S. aureus*

**Fig. 8:**
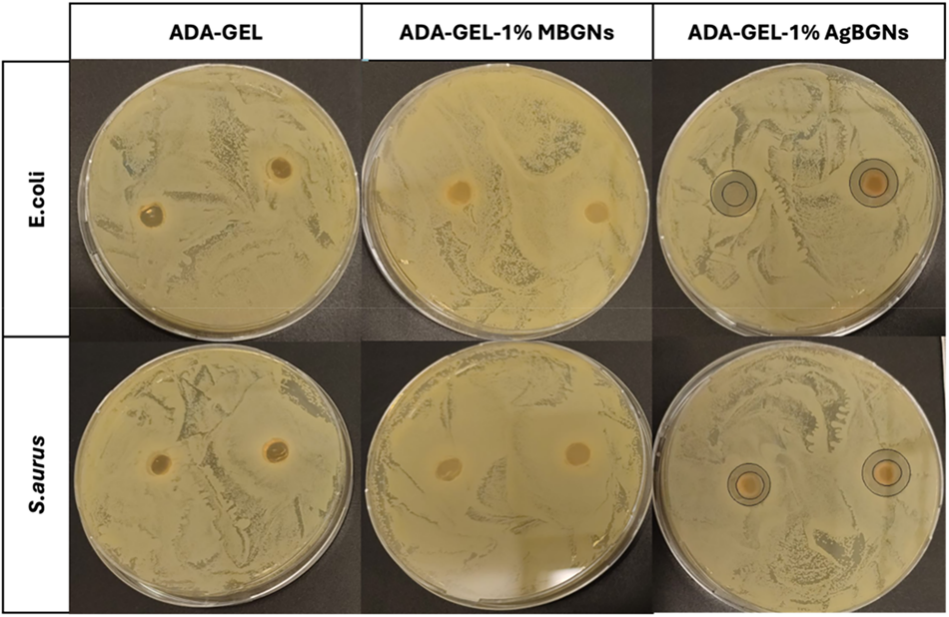
Antimicrobial activity of ADA-GEL and composite films with high concentration of nanoparticles against *E. coli* and *S. aureus*

The antibacterial activity of ADA-GEL hydrogels incorporating either AgBGNs or MBGNs nanoparticles was evaluated using the agar diffusion method. After 24 h of incubation, only the hydrogels containing 1% or 0.1% w/w AgBGNs exhibited a measurable zone of inhibition, as shown quantitatively in Fig. 6 and presented by the optical images in Figs. 7 and 8 for low and higher concentrations of nanoparticles, respectively. All other formulations, including those containing 0.1 and 1% MBGNs, and the control hydrogel without nanoparticles, showed no visible inhibition zones against both tested bacterial strains.

A consistent inhibition zone beyond the edge of the 0.1% Ag-doped hydrogel was observed in all three replicates, while no inhibition was detected around the control hydrogel.

### In vitro cytotoxicity

**Fig. 9:**
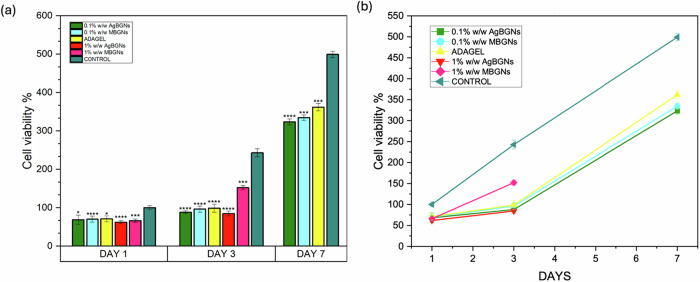
**a** Cell viability of MG-63 cells incorporated in pure ADA-GEL hydrogel films and ADA-GEL-NPs hydrogels with 0.1 and 1% (w/v) NPs variation after 1, 3, and 7 days of incubation. **b** Proliferation rates of MG-63 cells cultured in the different studied groups after 1,3, and 7 days. Values shown are mean and standard deviation (*n* = 3). *p < 0.05; ***p < 0.001; ****p < 0.0001

The metabolic activity of osteosarcoma MG-63 cells was assessed by the WST-8 assay after 1, 3, and 7 days of culture, in ADAGEL and in the composites with nanoparticle concentrations of 0.1% or 1% w/w. The data presented in Fig. 9a depict the cell viability for all studied groups, similar to that of the control group. The results are presented for the groups with a concentration 0.1% w/w for up to day 7, while for the groups with a higher concentration, the culture was studied up to day 3. Beyond this point, the results were deemed unreliable for the 1% w/w group, as these composite hydrogels had largely degraded after day 3, making the measurements at subsequent time points less reliable.

All experimental groups showed relatively high mitochondrial activity on day 1, with values ranging from approximately 61–71%. Samples containing 1% nanoparticles demonstrated slightly reduced viability compared to those with lower concentrations, likely due to increased ion release.

On day 3, all experimental groups, with the exception of composites with MBGNs 1%w/w, experienced similar growth rates as presented in Fig. 9b. Interestingly, by day 3, the MBGN-containing group exhibited higher viability compared to the other samples.

By day 7, the control group showed a nearly linear increase in proliferation throughout the incubation period (Fig. 9b). An increase in growth rates was observed in the lower-concentration samples, and their proliferation patterns beyond day 3 approached those of the control.

**Fig. 10:**
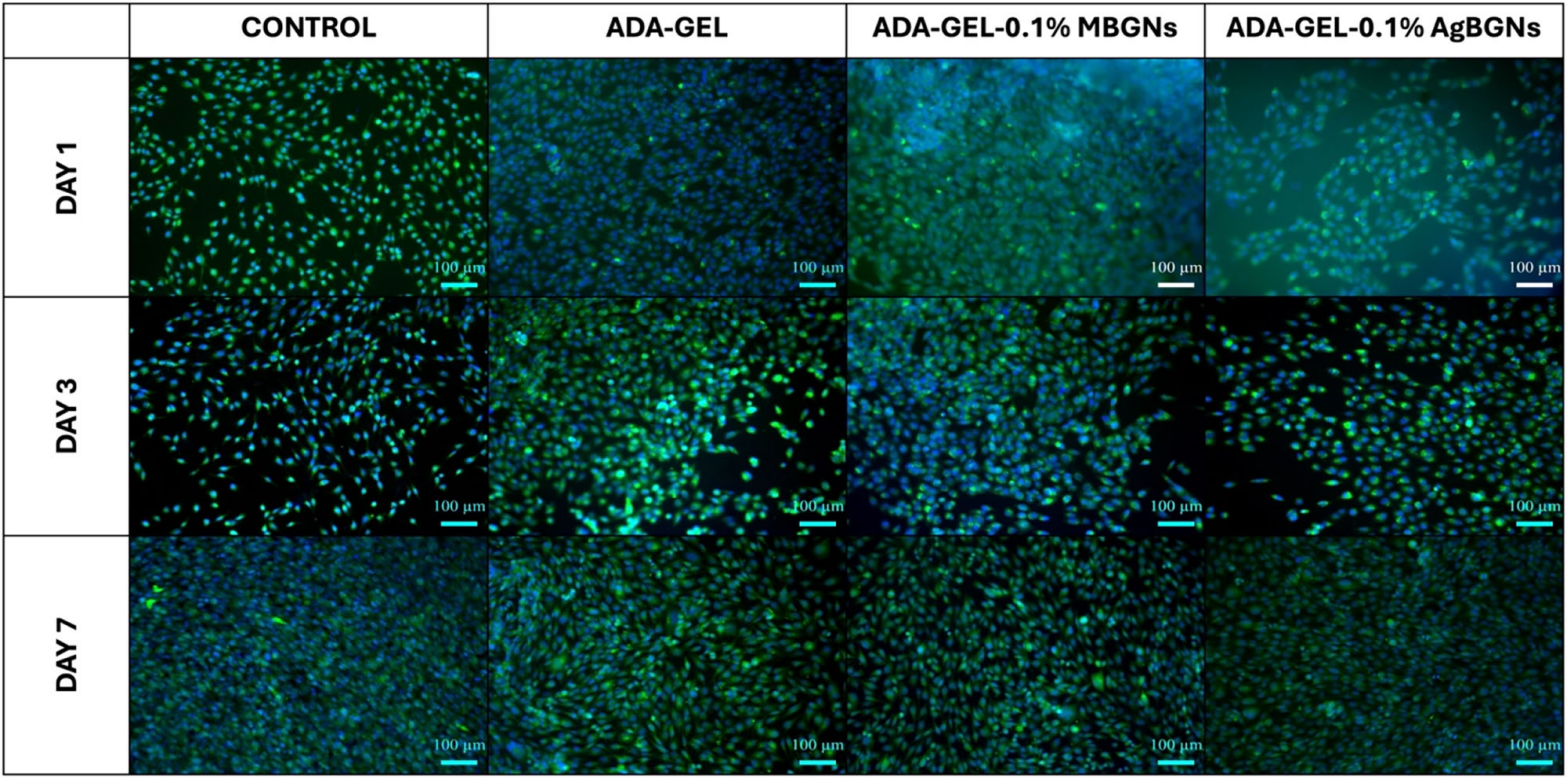
Fluorescence microscopy images of the composite hydrogel films after incubating for 1, 3, and 7 days with MG-63 cells. In control cells were cultured on tissue culture plate alone

The optical density was observed to increase over time. On days 1 and 7, cells assume elongated morphologies with extended filopodia (Fig. 10). Conversely, on day 3, the cells appear more rounded and clustered.

## Discussion

The observed spherical shape and nanoscale size of MBGNs and Ag-BGNs are in agreement with those of bioactive glass nanoparticles produced via the sol–gel method, as reported in the literature [10, 13]. Their amorphous structure, confirmed by XRD analysis, is crucial for bioactivity, enabling ion exchange and dissolution. FTIR results indicated that the glass network remained stable after adding silver, while the increased non-bridging oxygens suggest a higher potential for ion release, which is especially important for biological activity and antibacterial effects.

A major benefit of hydrogels is their high-water content and swelling ability, which enables them to absorb and hold large amounts of water [7, 23]. This property enables hydrogels to mimic natural tissues better. The porosity within the hydrogel matrix is essential for swelling, which occurs until the osmotic pressure from absorbing the surrounding solution balances with the elasticity of the polymer network [19, 24]. Adding nanoparticles increases free volume by disrupting the ideal alignment or entanglement of polymer chains, resulting in a looser polymer structure and greater solution uptake into the matrix [25]. Biodegradation benefits films as it supports the formation of new tissue. Based on average weight measurements, samples with Ag-BGNs showed a greater change in weight compared to other ADA-GEL-based hydrogels. This effect may be due to the structural and compositional effects of the added nanoparticles. In the case of Ag-BGNs, silver ions can disrupt the uniform crosslinking of the ADA chains. Unlike calcium ions, which form strong bidentate bonds with two carboxylate groups of ADA, creating a stable structure, silver ions might engage in weaker and less efficient coordination interactions with the polymer matrix. This can cause local differences in crosslink density and produce a more loosely organized hydrogel network, allowing for increased water absorption and swelling. Similarly, MBGNs, which are mainly composed of SiO₂ and CaO, add high surface area and porosity to the hydrogel system [26]. Their mesoporous structure can hold and absorb large amounts of water, contributing to the observed weight changes. Additionally, calcium ions released from MBGNs may support the ionic crosslinking process, although their diffusion through the hydrogel matrix might vary, leading to regions of uneven crosslinking. This uneven network formation can also impact the structural stability of the composite. Both Ag-BGNs and MBGNs may thus make the polymer network more flexible and hydrated. The structural flexibility caused by uneven crosslinking and high porosity reduces the stiffness of the hydrogel, but it could be advantageous for applications requiring soft and adaptable biomaterials.

Notably, in the case of ADA-GEL–AgBGNs hydrogel, the porous structure was well preserved after freeze-drying, showing a fine and interconnected morphology compared to ADA-GEL–MBGNs. Maintaining porosity after freeze-drying is highly valuable, especially for biomedical applications. In tissue engineering scaffolds, porous networks support cell adhesion, migration, and nutrient flow, aiding effective tissue regeneration [27]. In drug or ion release systems, open pores allow for controlled and sustained release of therapeutic ions like Ag⁺ and Ca²⁺, along with other bioactive molecules [25, 26]. Additionally, in regenerative medicine, a well-preserved pore structure closely mimics the natural extracellular matrix, creating a microenvironment that promotes cell growth and tissue integration [18, 28]. In AgBGN-containing hydrogels, maintaining porosity ensures continuous diffusion of Ag⁺ ions, enhancing antimicrobial effectiveness while preserving the scaffold’s overall biocompatibility.

Although the zone of inhibition of the 0.1% Ag-doped hydrogel was small (Fig. 6), its reproducibility and absence in the control support the conclusion that 0.1% Ag presents measurable antibacterial activity [29]. The observed increase in inhibition zone size with higher silver concentrations confirms the role of Ag in the glass structure of the incorporated AgBGNs mediates antibacterial activity. The detection of a measurable zone even at 0.1% suggests that the hydrogel facilitates ion release [30]. Several studies [31–33] consistently highlight the thicker peptidoglycan layer present in Gram-positive bacteria as a major factor in their higher resistance. This dense peptidoglycan wall acts as a barrier, limiting the penetration and intracellular uptake of silver ions and nanoparticles. In contrast, Gram-negative bacteria like *E. coli* possess a much thinner peptidoglycan layer, allowing easier diffusion of AgNPs and silver ions into the bacterial cell. The electrostatic interactions between the negatively charged bacterial cell membrane and the AgNPs facilitate rapid internalization in Gram-negative bacteria. Once inside, AgNPs disrupt vital cellular functions by interacting with proteins, DNA, and the cell membrane, ultimately leading to cell death [31].

The overall increase in MG-63 cell viability on day 1 indicates ongoing cell proliferation and confirms the biocompatibility of the hydrogels, even at higher particle concentrations. On day 3, the higher viability of the MBGN-containing group may be attributed to the accelerated degradation observed in this composite when seeded with cells. As documented in previous studies [34], higher degradation rates have been shown to facilitate cell proliferation, which correlates with the enhanced viability observed in this case. This degradation not only constrained the reliability of the measurements on days 3 and 7 but also hindered the preparation of samples for fluorescent imaging, as the films were unable to withstand staining or mechanical handling necessary for microscopy. The higher rate for the MBGN-containing composite group and the relatively lower growth rate for the AgBGNs-containing composites could also be attributed to the differences in ion release from the different compositions. While MBGNs release Si and Ca ions that are known to promote cell proliferation, silver ions may impact the mitochondria and induce mitochondrial swelling, causing slightly reduced viability [35, 36]. The proliferation patterns beyond day 3 (Fig. 9b), suggests that MG-63 cells quickly adapt to the different hydrogel composite samples, similar to their adaptation to the ADA-GEL matrix alone. The observed increase in optical density over time indicates both an increase in cell number and the overall metabolic activity of the cell population. Previous studies have demonstrated that, in 2D culture systems, cell proliferation closely correlates with mitochondrial activity [37]. The reduced cell viability observed for all studied groups, including ADAGEL alone compared to the control, suggests that this reduction cannot be solely assigned to the presence of nanoparticles. An explanation is that the hydrogels were directly crosslinked within the wells, potentially leading to incomplete crosslinking at the bottom of the gels. As previously documented, residual aldehyde groups are known to affect cell viability adversely and may even be cytotoxic [38]. Nevertheless, this effect was relatively minor and could be alleviated by prolonging the crosslinking duration [26, 27]. The elongated cell morphologies on days 1 and 7 indicate healthy attachment and spreading on the hydrogel surface. While the morphology of the cells on day 3 suggests reduced attachment and potential cellular stress, consistent with the mitochondrial activity data. Overall, the hydrogels demonstrate commendable biocompatibility and they effectively support cell adhesion and proliferation over time.

## Conclusions

In this study, multifunctional ADA-GEL hydrogel films with mesoporous bioactive glass nanoparticles (MBGNs) and silver-doped bioactive glass nanoparticles (Ag-BGNs) were successfully developed. The findings show that adding nanoparticles notably changed the physicochemical and biological properties of the ADA-GEL matrix.

MBGNs enhanced swelling behavior, supported degradation, and provided additional calcium ion release, which contributed to improved structural stability and osteogenic potential. Simultaneously, AgBGNs introduced strong antibacterial properties against both Gram-positive and Gram-negative bacteria, with observed activity even at low concentrations, without significantly compromising biocompatibility. Importantly, all hydrogel formulations supported the adhesion, proliferation, and metabolic activity of MG-63 cells, confirming their suitability as bioactive and cytocompatible scaffolds.

The hydrogels showed improved printability, as low nanoparticle concentrations (0.1% w/v) helped enhance filament fidelity and structural accuracy during extrusion-based 3D printing. These results emphasize the versatility of ADA-GEL-based nanocomposites as customizable biomaterials that blend the extracellular matrix–mimicking qualities of natural polymers with the regenerative and protective capabilities of advanced nanoparticles like the ones studied here (MBGNs and AgBGNs).

Overall, ADA-GEL/MBGN and ADA-GEL/Ag-BGN composite hydrogels are promising candidates for biomedical applications, where bioactivity, mechanical stability, and antibacterial action are essential. Future research will focus on optimizing crosslinking methods, assessing long-term stability, and testing their performance in more complex in vitro and in vivo models to further support their clinical use.

## Data Availability

The datasets generated during and/or analyzed during the current study are available from the corresponding author on reasonable request.
